# Молекулярные онкологические консилиумы и тераностика

**DOI:** 10.14341/probl13220

**Published:** 2022-12-20

**Authors:** П. О. Румянцев

**Affiliations:** Группа компаний «Мой медицинский центр»

**Keywords:** молекулярный онкологический консилиум, онкология, тераностика, молекулярный профиль опухоли

## Abstract

Клиническая онкология сегодня проходит период беспрецедентных изменений. Таргетная терапия, а в последующем иммунотерапия, революционизировали клиническое течение и исход заболевания у многих пациентов с солидными злокачественными новообразованиями (ЗНО). Клиническая онкология неотделима от молекулярной онкологии, развитие которых взаимосвязано. Молекулярно-обоснованный выбор наиболее прецизионной, эффективной и наименее токсичной схемы противоопухолевой терапии является крайне актуальной клинической задачей, особенно при жизнеугрожающих и резистентных к иным видам лечения случаям ЗНО. Современные технологии геномных и постгеномных исследований, а также методы молекулярной визуализации (позитронная и однофотонная эмиссионная компьютерная томография (ПЭТ и ОФЭКТ)) позволяют не только оценивать метаболический и рецепторный статус очагов опухоли, но и подбирать как ключ к замку оптимальную терапевтическую тактику. В клинической практике онкологии все чаще возникает необходимость в молекулярных онкологических консилиумах (МОК). Опубликованный опыт реальной клинической практики применения рекомендованных МОК режимов лечения на основе молекулярного гено-транскриптомного профиля опухоли свидетельствует о лучших результатах безрецидивной и общей выживаемости пациентов в сравнении с лечением, назначаемым врачом без учета молекулярного профиля опухоли. Необходимы дальнейшее накопление опыта и проведение рандомизированных контролируемых клинических испытаний для более основательных и доказательных выводов. Однако нет никаких сомнений, что МОК является мощнейшим инструментом развития прецизионной персонализированной онкологии.

## Abstract (en)

Clinical oncology is currently undergoing a period of unprecedented change. Targeted therapy, and subsequently immunotherapy, has revolutionized the clinical course and outcome of many patients with solid cancer. Clinical oncology is inseparable from molecular oncology, the development of which is interconnected. Molecular tumor research proposes the most precise, effective and lesser toxic antitumor therapy regimen is an extremely urgent clinical task, especially in life-threatening and resistant to other types of treatment cases of cancer. Modern technologies of genomic and postgenomic studies, as well as molecular imaging methods (positron and single photon emission computed tomography, PET and SPECT, respectively) make it possible not only to assess the metabolic and receptor status of tumor foci, but also to select the optimal therapeutic tactics as a key to the lock. In the clinical practice of oncology, there is an increasing need for molecular tumor board (MTB). Published real clinical experience with MTB-recommended treatment regimens based on the molecular geno-transcriptomic profile of the tumor indicates better relapse-free and overall patient survival compared to treatment prescribed by a physician without taking into account the molecular profile of the tumor. More experience is needed and randomized controlled clinical trials are needed for more solid and evidence-based conclusions. However, there is no doubt that the MTB is a powerful tool for the development of precision personalized oncology.

Злокачественные новообразования (ЗНО) обладают различным потенциалом опухолевой диссеминации, которая может наблюдаться на любой стадии опухоли. С одной стороны, это требует совершенствования стадирования, прогнозирования, оценки эффективности лечения и предикции ответа опухоли на терапию. Современные технологии геномных и постгеномных исследований, а также методы молекулярной визуализации (позитронная и однофотонная эмиссионная компьютерная томография — ПЭТ и ОФЭКТ) позволяют не только оценивать метаболический и рецепторный статус очагов опухоли, но и подбирать как ключ к замку оптимальную терапевтическую тактику. Такой подход в современной биомедицине получил название тераностика (ТЕРАпия ⬄ диагНОСТИКА), а направление по поиску молекулярно-генетических предикторов — радиогеномика.

В свою очередь, опухолевая диссеминация требует своевременной и максимально эффективной противоопухолевой системной терапии (химиотерапия, таргетная, иммунотерапия, радиофармацевтическая), часто в сочетании с локальными (хирургия, радиотерапия, брахитерапия и др.) методами лечения.

Первый опыт применения химиотерапии ЗНО насчитывает историю с 1940-х гг. и начинался с использования одного химиопрепарата, который в лучшем случае вызывал транзиторную ремиссию опухоли и развитие резистентности. В свою очередь, это вызвало интерес к новым химиотерапевтическим комбинациям, в том числе для преодоления резистентности опухоли. И сегодня наиболее эффективные химиотерапевтические схемы являются комбинацией препаратов с различными механизмами действия, и по этой причине механизмы развития резистентности к таким схемам не перекрываются.

Обнаружение в 1980-х гг. онкогенов, индуцирующих развитие злокачественных опухолей, предоставило возможность заглянуть в молекулярные механизмы канцерогенеза, что, в свою очередь, позволило разработать принципиально новые поколения таргетных противоопухолевых препаратов, которые действовали избирательно на активированные сигнальные пути в раковых клетках.

Хотя это и было огромным достижением, появилась новая головоломка: как наилучшим образом подобрать комбинацию из огромного числа терапевтических агентов для получения наиболее эффективного, прецизионного и длительного, устойчивого ответа опухоли по аналогии с подбором эффективной комбинации препаратов, воздействующих на различные клеточные мишени и позволяющих добиваться долговременного контроля болезни у ВИЧ-инфицированных пациентов.

Широкое применение в последнее время технологий секвенирования следующего поколения (Next-generation sequencing, NGS) и развитие биоинформационных ресурсов закономерно повлекли за собой формирование практики молекулярных онкологических консилиумов (МОК). NGS запустило эру индивидуального генотипирования опухоли с яркими примерами успешной конгруэнтности между терапевтической актуальностью генетических повреждений и таргетными препаратами, которые делают клинический случай препарат-таргетируемым.

Методы молекулярной визуализации (ПЭТ/КТ, ПЭТ/МРТ, ОФЭКТ/КТ), которые сегодня широко вошли в клиническую практику клинической онкологии и эндокринологии, продолжая активно развиваться, открывают новые горизонты в раннем выявлении, стадировании, дифференциальной диагностике и тераностике онкологии и онкоэндокринологии. В сочетании с молекулярно-генетической диагностикой они позволяют неинвазивно in vivo визуализировать очаги опухоли в теле пациента, охарактеризовать их метаболический и рецепторный статус, обеспечить прицельность биопсии и радиотерапии, радионавигацию при хирургии, осуществлять персонализированный отбор на радиофармацевтическую терапию, обеспечить оценку ответа на лечение и раннее выявление рецидива опухоли.

Клиническая онкология сегодня проходит период беспрецедентных изменений. Таргетная терапия, а в последующем — иммунотерапия революционизировали клиническое течение и исход заболевания у многих пациентов с солидными ЗНО. Клиническая онкология неотделима от молекулярной онкологии, развитие которых взаимосвязано. Молекулярно-обоснованный выбор наиболее прецизионной, эффективной и наименее токсичной схемы противоопухолевой терапии является крайне актуальной клинической задачей, особенно при жизнеугрожающих и резистентных к иным видам лечения случаям ЗНО.

В каждом конкретном клиническом случае прежде всего стоит определиться с целью и составом МОК. Целью МОК является оценка выбора с позиции накопленной базы знаний доказательной медицины и с учетом результатов молекулярно-генетических исследований оптимальной тактики ведения конкретного клинического случая. Правильность гистологической верификации стадии опухоли в соответствии с действующей классификацией, группа клинического риска рецидива/прогрессирования опухоли и многое другое являются предметом обсуждения и междисциплинарного консенсуса в рамках МОК. Клиническая картина, данные лабораторных и инструментальных методов обследования, интерпретируемые с учетом накопленного доказательного мирового опыта, в том числе атипичных вариантов клинического течения заболевания, не должны противоречить результатам морфологических и молекулярно-генетических исследований. Скорее, наоборот, они должны взаимодополнять друг друга, позволяя предсказывать наиболее вероятное «поведение» опухоли и вероятные клинические сценарии.

Очень важно понимать, сколько есть времени на принятие решения по тактике ведения, перепроверке конфликтующих данных, уточнение отдельных параметров опухоли, биомаркеров (радиомных, морфологических, биохимических) и динамики процесса. Немаловажной является оценка медико-экономических параметров, которые складываются из расчета прогнозируемых затрат на стоимость диагностики, лечение и реабилитацию, их вероятного терапевтического и токсического эффекта, продления длительности и качества жизни пациента.

Результаты молекулярного анализа опухоли могут включать:

Заключение МОК должно включать интерпретацию результатов молекулярно-генетической и транскриптомной панелей, а также доказательные рекомендации по наиболее оптимальным линиям терапии и вероятности ответа на лечение с информацией по соответствующим клиническим испытаниям и программам открытого доступа, если таковые проводятся в настоящее время или планируются. МОК также могут служить важным образовательным инструментом в университетских клиниках с накоплением хрестоматийных клинических случаев или ситуационных задач, воспитывающих традиции «клинико-молекулярных» консультаций. В соответствии со стандартами и рекомендациями Американской ассоциации молекулярных патологов, Общества клинических онкологов и коллегии патологоанатомов отчеты МОК должны включать название изучавшихся генов и их локусов; экзонов или горячих точек; интронных последовательностей, вовлеченных в транслокации с генерацией слияний генов; покрытие генетических полей для оценки мутационной нагрузки; а также тестированное число и тип микросателлитов для оценки микросателлитной нестабильности.

## КАКИЕ СЛУЧАИ ОПУХОЛЕЙ ПОДХОДЯТ ДЛЯ ОБСУЖДЕНИЯ НА МОК?

В первую очередь на МОК должны обсуждаться те случаи опухоли, терапия которых в соответствии с клиническими рекомендациями и доказательным опытом неизвестна или недостаточно эффективна. Это столь же актуально при неоднозначном гистологическом заключении или несоответствии его клинической картине и течению болезни. По данным одного из обзоров, включавшего 440 случаев ЗНО с четким гистологическим заключением, необходимость в МОК чаще всего возникала при саркомах (21,4%), раке молочной железы (20%), опухолях головного мозга (15,5%), онкогинекологии (14,1%), раке легкого (7,3%) и колоректальном раке (6,4%).

Важно понимать, что для обсуждения на МОК потенциально подходят все типы и локализации ЗНО, причем гистологический тип опухоли не является дискриминирующим критерием. Более того, редкие (орфанные) ЗНО, которые совокупно представляют примерно четверть всех случаев, гораздо более актуальны для обсуждения на МОК, чем часто встречающиеся карциномы. Молекулярный портрет опухоли должен рассматриваться как обязательная интегративная часть гистологического диагноза при ЗНО, у которых отсутствуют стандартизованные рекомендации по терапии.

## КАКИЕ МОЛЕКУЛЯРНО-ГЕНЕТИЧЕСКИЕ АНАЛИЗЫ НЕОБХОДИМО ПРОВОДИТЬ ДЛЯ ОБСУЖДЕНИЯ НА МОК?

По данным анализа публикаций, существует множество вариантов молекулярных анализов. В большинстве случаев (57,4%) для обсуждения на МОК выполнялось таргетное мультигенное секвенирование следующего поколения (Targeted Multigene Next Generation Sequencing, MG-NGS), которое дополнялось полноэкзомным секвенированием (Whole-Exome Sequencing, WES) в 16,4%, секвенированием РНК (RNA sequencing) — в 13,1%, матричной сравнительной геномной гибридизацией (Array Comparative Genomic Hybridization, aCGH) — в 4,9%, полногеномным секвенированием (Whole Genome Sequencing, WGS) и секвенированием по Сэнгеру (Sanger sequencing, SS) — по 3,3%, анализом мРНК (mRNA) — в 1,6% случаев. Наиболее подходящим анализом с целью обнаружения полезных в практическом смысле генетических нарушений является таргетное NGS наиболее важных рак-ассоциированных генов, в идеале в сопряжении с анализом на клинически релевантные генетические фьюжины (например, слиянием киназных генов). Этот анализ широко распространен и связан с относительно коротким временем на секвенирование и посильной биоинформатикой.

## КАКИЕ СПЕЦИАЛИСТЫ ДОЛЖНЫ ОБЯЗАТЕЛЬНО ПРИНИМАТЬ УЧАСТИЕ В МОК? КАКИЕ СПЕЦИАЛИСТЫ МОГУТ ПОНАДОБИТЬСЯ ДОПОЛНИТЕЛЬНО?

МОК – важнейший инструмент побуждения «клинико-молекулярных» обсуждений и поощрения коммуникации между коллегами различных специальностей, что значительно ускоряет развитие персонализированной медицины. Как минимум в составе МОК обязательно должны быть следующие ключевые специалисты: клинические онкологи (по данным литературы в 100% МОК), патологоанатомы (90,6%). Также велика роль генетиков (68,7%), особенно при обсуждении вопросов в отношении герминальных мутаций, биоинформатиков (37,5%) и молекулярных биологов (25%), особенно при интерпретации массивных наборов данных в результате использования технологий NGS, например при WGS. Включение в состав МОК специалистов лучевой диагностики и эндоскопии (при необходимости) также очень желательно. Менее часто участвуют фармакологи (21,9%) и специалисты по биоэтике (9,4%), подключение которых особенно актуально при использовании экспериментальных препаратов. В 12,5% в МОК включались клиницисты-исследователи со знанием «молекулярной медицины».

## КАКОВ ОПТИМАЛЬНЫЙ СРОК ПРОВЕДЕНИЯ МОК?

Во многом этот период определяется индивидуальной клинической ситуацией. В реальной клинической практике, по данным литературы, данный срок варьирует от 12 до 86 дней и в среднем составляет 38 дней. Минимальный период ожидания решения МОК в клинической практике, по данным литературы, был в среднем 16 дней. Компромиссный срок проведения МОК с учетом клинической необходимости и времени на проведение масштабных молекулярных исследований находится в пределах 28 дней (4 нед). Разумеется, чем этот период меньше, тем выше шансы на изменение клинической ситуации к лучшему путем назначения эффективной таргетной терапии.

Чаще всего причиной задержки молекулярных исследований являются организационные, технические и «человеческие» проблемы, связанные с возможностью получения парафиновых блоков и их транспортировкой в лабораторию, низкое качество тканевого материала в парафине (поздняя передача в лабораторию патанатомии макропрепарата, передерживание его в формалине, использование чистого формалина (а не забуференного раствора), дефекты проводки и заливки, низкое качество парафина), а также его контаминация на этапе пробоподготовки. На качество пробоподготовки в патологоанатомической лаборатории невозможно повлиять обратным порядком, поэтому столь важно оперировать или выполнять биопсию в тех медицинских учреждениях, которые имеют высокую валидированную репутацию, в том числе по части качества парафиновых блоков и морфологической верификации. Помимо этого, важнейшим фактором является контроль взятия на молекулярные исследования клеточного материала из опухоли. Это осуществляется путем контролируемого взятия биологического материала под контролем гистологической верификации источника.

Отличным способом уменьшить срок выполнения молекулярно-генетических исследований является так называемый «рефлекс-тест» (reflex test). Этот способ заключается в том, что молекулярно-генетический тест назначается врачом-патологоанатомом уже при изучении гистологических стеклопрепаратов, когда срабатывает «рефлекс» выяснить молекулярный профиль определенного гистологического паттерна (например, рак поджелудочной железы с медуллярным гистологическим строением, который обычно ассоциирован с микросателлитной нестабильностью).

В случае наличия биобанка молекулярный анализ может быть ускорен и стать более качественным с учетом того, что криоконсервированный образец опухоли предпочтительнее для молекулярно-генетических исследований, чем парафиновый блок. Идентификация опухолевой природы криообразца осуществляется при помощи гистологического исследования участка, откуда взят образец опухоли для криоконсервации.

Довольно сложно оценивать рентабельность молекулярно-ориентированной терапии в онкологии, так как «лавры» всегда достаются таргетному препарату или их комбинации. Сама по себе активность МОК (в основном трудозатраты) составляет не более 5% стоимости молекулярной диагностики, а в стоимости общих лечебно-диагностических затрат занимает около 0,3%. Чаще всего стоимость современных таргетных препаратов намного превышает затраты на молекулярно-генетические исследования и МОК, но без выполнения последних бывает невозможно переломить негативный клинический сценарий.

Также молекулярно-генетическое тестирование МОК открывает новые горизонты для включения пациентов в клинические исследования и программы открытого доступа фармкомпаний по новым таргетным препаратам. В отсутствие таковой возможности пациенты, социальные службы и фонды могут инициативно на основании рекомендаций МОК приобретать зарегистрированные в стране противоопухолевые препараты, а также ввозить из-за рубежа по индивидуальным жизненным показаниям.

Включение в формализованные (on-label) клинические испытания во всем мире является предпочтительным путем получения таргетной терапии как минимум по двум причинам:

При анализе мутаций могут быть обнаружены герминальные онкомутации, требующие проведения генетического скрининга на предмет носительства мутации среди кровных родственников пробанда, что, в свою очередь, позволяет улучшить раннюю диагностику опухоли и выполнять, к примеру, превентивное хирургическое лечение, если таковое осуществимо.

Мультидисциплинарная парадигма МОК позволяет различным специалистам улучшить коммуникацию в интересах не только обсуждаемого, но и многих других пациентов. Конструктивная дискуссия диагностов (радиологов, рентгенологов, патоморфологов, специалистов лабораторной диагностики) с лечебниками (хирургами, радиотерапевтами, химиотерапевтами, специалистами радионуклидной терапии) по поводу предметного клинического случая повышает потенциал современной биомедицины в конкретном медицинском учреждении, учит командной работе и создает благоприятные условия для развития научно-практического сотрудничества. Более того, накопление информации и обмен опытом стандартизируют работу МОК, расширяют ее компетенции и производительность, увеличивают доказательный опыт применения on-label и off-label таргетных препаратов, диагностических технологий, в том числе целенаправленного геномного и постгеномного секвенирования.

В дополнение к опухолевым клинико-морфологическим факторам выяснение молекулярного профиля опухоли может предоставлять возможности использования более информативных диагностических методов и коррекции лечебной стратегии на более эффективную. Накопление «молекулярно-клинического» опыта и пополнение доказательной базы требуют совершенствования технологий и принципов, непрерывно обогащаемых собственным и зарубежным опытом.

В качестве примеров клинической значимости молекулярного профиля опухоли и роли молекулярной визуализации (ПЭТ/КТ, ОФЭКТ/КТ) в планировании стратегии ведения пациента можно привести опухоли эндокринной системы, например, болезнь Кушинга и рак щитовидной железы. При болезни Кушинга (опухоль гипофиза, продуцирующая АКТГ) агрессивность клинического течения значительно варьирует. По данным полноэкзомного секвенирования частота мутаций в клетках опухоли варьирует от 20 до 60%. При более углубленных молекулярных исследованиях обнаружены дополнительные мутации в гене глюкокортикоидного рецептора NR3C1, а также онкогена BRAF, деубиквитиназа-кодирующем гене USP48, гене TP53, которые встречались гораздо реже. Более того, в дальнейшем было выявлено, что кортикотрофные опухоли с диким и мутантным типами гена USP8 имели совершенно различные транскриптомные профили и как результат обладали разной гормональной активностью и клинической агрессивностью. При недифференцированной (анапластической) карциноме щитовидной железы, крайне агрессивной ЗНО, наличие мутации BRAF в клетках опухоли позволяет успешно применять комбинацию селективных BRAF и MEK-киназных ингибиторов для системной терапии болезни. Визуализация очагов медуллярного рака щитовидной железы (нейроэндокринной опухоли) с помощью соматостатин-рецепторной сцинтиграфии (ОФЭКТ/КТ с тектротидом или ПЭТ/КТ с 68Ga-DOTA-TATE/NOC) позволяет не только улучшить стадирование опухоли, но также является основанием для коррекции терапевтической стратегии добавлением в алгоритм лечения аналогов соматостатина и пептид-рецепторной радионуклидной терапии (177Lu-DOTA-TATE, LutatheraTM).

В идеале терапевтический выбор должен опираться на наличие таргетированных драйверных мутаций, анализируемых целенаправленно, или агностических1 (выявленных при поисковых исследованиях) молекулярных биомаркеров, что сегодня составляет авангард прецизионной персонализированной онкологии, эндокринологии и других областей современной биомедицины. Практически значимые агностические молекулярные мишени стали предиктивными биомаркерами для соответствующих таргетных препаратов, эффективных без относительности к локализации и гистологии карциномы.

Разработаны дизайны «корзинных» (Basket) и «зонтичных» (Umbrella) клинических исследований для ускорения разработки новых таргетных противоопухолевых препаратов. В связи с жесткими критериями включения в такие исследования набор пациентов был низким, что отчасти послужило поводом для широкого внедрения технологий прецизионной молекулярной онкологии в клиническую практику по всему миру. В результате резко возросло количество онкологических пациентов, являющихся потенциальными кандидатами на изучение молекулярного профиля опухоли на фоне растущего числа потенциальных терапевтических молекулярных мишеней.

Сложность интерпретации молекулярных данных также увеличивается. Адекватно транслируемая и интерпретируемая информация о комплексе с генетическими и транскриптомными особенностями опухоли предоставляет клиницистам новые возможности моделирования индивидуализированной лечебно-диагностической стратегии. Это, разумеется, коренным образом меняет привычный функционал и потенциал возможностей традиционных онкологических консилиумов, повышая их персонализированную молекулярно-генетическую осведомленность.

МОК может оказаться полезен в выборе наиболее подходящего биологического образца для молекулярного анализа, например, гистологического блока или жидкостной биопсии крови, технологий молекулярно-генетических анализов и преимуществ конкретных тестов. Жидкостная биопсия крови с целенаправленной оценкой профиля геномных нарушений позволяет обеспечить мониторинг уже известных препарат-таргетируемых драйверных мутаций.

В клинической практике сегодня применяется жидкостная биопсия крови для обнаружения драйверных мутаций в циркулирующей опухолевой ДНК у пациентов с раком легких. Данная технология активно исследуется и при других солидных ЗНО.

МОК особенно важен у пациентов с распространенными ЗНО, а именно:

Опубликованный опыт реальной клинической практики применения рекомендованных МОК режимов лечения на основе молекулярного генно-транскриптомного профиля опухоли свидетельствует о лучших результатах безрецидивной и общей выживаемости пациентов в сравнении с лечением, назначаемым врачом без учета молекулярного профиля опухоли. Необходимы дальнейшее накопление опыта и проведение рандомизированных контролируемых клинических испытаний для более основательных и доказательных выводов. Однако ни у кого из специалистов нет сомнений, что МОК является мощнейшим источником развития прецизионной персонализированной медицины. Необходимо продолжить работу по стандартизации исовершенствованию принципов работы МОК, стимулировать создание и пополнение мультидисциплинарных банков данных пациентов, многоцентровые исследования, обмен и тиражирование успешного опыта.

Интеграция научной и клинической практики ускоряет комплексное и прикладное исследование генетики рака, а также приближает создание и исследование новых противоопухолевых препаратов и их действенных комбинаций. Созданием эффективной сетевой системы МОК позволит преодолеть неоднородность критериев выбора пациентов для молекулярных исследований, совершенствовать на более высоком доказательном уровне технологии геномных и транскриптомных анализов, а также оптимизировать выбор терапевтической и диагностической стратегии для каждого пациента.

Важно обеспечить транспарентность работы и кросс-валидацию результатов молекулярного тестирования в целях обеспечения надлежащего качества исследований, особенно это касается анализов на основе NGS, современных программ обучения и обмена опытом.

При обсуждении на МОК лечебной тактики вполне уместны и поощряются дискуссии в отношении накопленного клинического опыта, биоинформатики, преаналитических и аналитических моментов, включая выбор типа биологического материала (ткань или жидкостная биопсия), молекулярно-генетических анализов (полногеномный или целенаправленный) и т.п.

## КРИТЕРИИ ВЫБОРА БИОЛОГИЧЕСКИХ ОБРАЗЦОВ И ВИДОВ МОЛЕКУЛЯРНЫХ ИССЛЕДОВАНИЙ

Выбор биологического образца является важнейшим вопросом для информативности последующих молекулярных анализов. Прежде всего очень важно обсудить на МОК, какой именно биоматериал (цитологический или гистологический) является наиболее информативным и будет направлен на молекулярные исследования. В эру прецизионной медицины количество биомаркеров для диагностических, предиктивных и прогностических целей постоянно растет. Однако в некоторых клинических случаях имеется возможность получения лишь небольшого количества биоматериала (например, при пункции под УЗИ-, МСКТ- или ПЭТ/КТ-контролем). Для молекулярных анализов, как правило, достаточно небольшого количества биоматериала, но необходимо обеспечить полноценную пригодность и сохранность извлеченного биопсийного образца для молекулярных исследований, особенно если объем биообразца небольшой. Рекомендуется изначально озаботиться обеспечением надлежащей пробоподготовки (недопущение контаминации, надлежащая маркировка, хранение в контейнере с забуференным раствором формалина, надлежащие условия транспортировки и хранения, качество консервации, минимизация периода доставки в лабораторию для молекулярных исследований). Все этапы пробоподготовки должны выполняться квалифицированным и опытным персоналом, согласованно и ответственно. Патологоанатомы играют ключевую роль, экспертную и координирующую, так как морфологическая верификация опухоли и той ее части, которая направляется на молекулярное исследование, определяет качество и точность последнего. Специализирующиеся на молекулярных исследованиях патологи могут оказать помощь в решении случайных или систематических проблем, связанных с дефектами пробоподготовки. Поэтому активное участие патологоанатома в составе МОК крайне желательно. Первичная морфологическая оценка биологического образца необходима для:

По существу патологоанатом должен оценить качественную и количественную адекватность тканевых образцов, особенно фокусируя внимание на пределах детекции молекулярных методов и нижнем пороге содержания нуклеиновых кислот в образце для анализа во избежание ложнонегативных или ненадежных результатов молекулярных исследований. Эти данные должны быть включены в отчет по молекулярным исследованиям для МОК. Более того, на МОК должны рассматриваться вопросы диагностической приоритетности цитологических и гистологических образов, а именно:

Жидкостная биопсия является валидной альтернативой молекулярному анализу, что особенно актуально при невозможности получения образца опухолевой ткани или его низком качестве. Жидкостная биопсия может содержать различные опухоль-содержащие жидкости, включающие циркулирующую опухолевую ДНК (цоДНК), опухолевые клетки и экзомы. Анализ цоДНК в плазме крови является наиболее широко используемой альтернативой опухолевой ткани для изучения генотипа солидных опухолей. После многочисленных попыток определения мутаций EGFR при немелкоклеточном раке легкого чувствительность и специфичность определения цоДНК методом NGS были значительно повышены. В связи с неинвазивностью и хорошей воспроизводимостью (стандартная процедура взятия образца венозной крови) жидкостная биопсия имеет важное значение при динамическом мониторинге опухоли, а также для преодоления проблем опухолевой гетерогенности и определения минимальной остаточной болезни (MRD, Minimal Residual Disease). C другой стороны, жидкостная биопсия крови не обладает еще достаточной чувствительностью и имеет высокий риск ложноотрицательных результатов. Метод жидкостной биопсии крови еще больше, чем биопсия ткани опухоли, зависит от качества преаналитической подготовки, начиная от процедуры забора образца крови, центрифугирования, экстракции ДНК и ее хранения. Жидкостная биопсия крови является чрезвычайно перспективным и бурно развивающимся направлением молекулярной онкологии, а ее роль будет только расти в ближайшем и отдаленном будущем.

## ЗАКЛЮЧЕНИЕ

Клиническая эффективность МОК определяется оперативностью и результативностью оптимального комплекса диагностических мероприятий и терапевтических решений на их основе, позволяющих улучшать выживаемость и качество жизни онкологических пациентов. Важными составляющими успешности в работе МОК являются: (а) получение надлежащего качества и количества биопсийного материала; (б) снижение уровня преаналитических ошибок, внедрение и стандартизация технологий тканевого сэмплинга/анализа; (в) информационная технологическая инфраструктура, позволяющая осуществлять оперативную и удобную для работы МОК инфраструктуру на многоцентровом уровне, а также медицинские банки данных (современные клинические регистры пациентов) для доказательного анализа эффективности и безопасности лечения большого числа клинических случаев; (г) непрерывное повышение эффективности и качества медицинской помощи путем совершенствования тераностики на основе «молекулярно-навигационных» технологий. Прецизионная медицина, особенно тестирование опухолевой ткани на наличие драйверных мутаций с помощью NGS с целью улучшения терапии, является огромным преимуществом в лечении рака и рассматривается как оптимальная опция персонализированной медицины.

## ДОПОЛНИТЕЛЬНАЯ ИНФОРМАЦИЯ

Источники финансирования. Работа выполнена по инициативе автора без привлечения финансирования.

Конфликт интересов. Автор декларирует отсутствие явных и потенциальных конфликтов интересов, связанных с содержанием настоящей статьи.

Участие авторов. Автор одобрил финальную версию статьи перед публикацией, выразил согласие нести ответственность за все аспекты работы, подразумевающую надлежащее изучение и решение вопросов, связанных с точностью или добросовестностью любой части работы.

1. Агностический — происходит от древнегреческого ἄγνωστος — «не основанный на уверенном знании». Агностический принцип применяется для поиска и анализа статистическими методами множества молекулярных мишеней. Осуществляется в целях обнаружения новых биомаркеров после изучения совокупности всех доступных генетических вариантов нарушений (мутаций) с интересующими признаками.
